# A replication of the relationship between elderly suicides rates and elderly dependency ratios: cross-national study

**DOI:** 10.5249/jivr.v2i1.53

**Published:** 2010-01

**Authors:** Ajit Shah

**Affiliations:** ^a^International School for Communities, Rights and Inclusion, University of Central Lancashire, Preston and Consultant Psychiatrist, West London Mental Health NHS Trust, London, United Kingdom.

## Abstract

**Background:**

A positive correlation between elderly dependency ratios and elderly suicide rates has been observed using one-year cross-sectional data on elderly suicide rates.

**Methods:**

A cross-national study designed to replicate this positive correlation between elderly dependency ratios and elderly suicide rates was undertaken by: (i) using one-year average of five years data on suicide rates; and (ii) using more recent data on both elderly suicide rates and elderly dependency ratios. Data on elderly suicide rates, and the total number of elderly and young people was ascertained from the World Health Organization website.

**Results:**

The main findings were of significant positive correlations between elderly dependency ratios and suicide rates in both sexes in both the elderly age-bands (65-74 years and 75+ years).

**Conclusions:**

The replication of the positive correlations between elderly dependency ratios and elderly suicide rates by using one-year average of five years data on suicide rates suggests that this relationship is robust and accurate.

## Introduction

Suicide rates among non-white Americans,^1^ Indians,^2,3,4^ Arabs in Jordan,^5^ men in Kuwait,^6^ Malays in Singapore,^7^ Indian immigrants to the United Kingdom,^8,9^ and in some east European countries^10^ decline with increasing age.

Traditionally, in these societies the elderly are respected, held in high esteem and live in closely knit families, and this offers protection against loneliness and despair, which otherwise may lead to suicide.^11^ These factors may also explain the low elderly suicide rates in Thailand^12^ and among Malays in Singapore.^13^ A similar hypothesis may also explain high suicide rates among the elderly in Japan,^14,15^ Hong Kong,^16^ China^17,18^ and Taiwan^19^ because they have lost their traditional role in the family. Additional evidence supporting this, from Hong Kong and China, includes: mismatch between the traditional dependence of elderly on their children for emotional and financial support and their children’s ability to provide this;^17,18,20^ the traditional expectation of the elderly to live with their children or grand-children not being met;^20^ the greater effect on the elderly of their children’s negative attitudes;^20^ and, the migration of children to urban areas or to other countries.^16,17^ Lower elderly suicide rates are associated with reduction in the number of caregivers,^13,20,22^ larger household size,^13,23^ greater proportion of extended family households^23^ and lower proportion of single family households.^23^

A recent cross-national study reported a positive correlation between elderly suicide rates and elderly dependency ratios (the ratio of people over the age of 65 years to people under the age of 65 years).^22^ This finding was primarily explained by speculating that presence of greater number of younger people being available to care for older people ultimately leading to reduction in elderly suicide rates based on the cultural explanations described above. ^22^

 However, this study used only one-year cross-sectional data on elderly suicide rates and suicide rates can randomly fluctuate year on year.^24^ Therefore, in order to replicate the findings of this earlier study, the relationship between elderly suicide rates and elderly dependency ratios was examined: (i) by using a one-year average of five years data on elderly suicide rates; and (ii) using the latest available (and, therefore, a more recent) data set on elderly suicide rates.

## Methods

Data on elderly suicide rates for males and females in the age-bands 65-74 years and 75+ years were ascertained from the World Health Organisation (WHO) website (http://www.who.int/whosis/database/mort/table1.cfm). For a small number of countries only the raw figures for the number of suicides were available from the WHO website. Suicide rates for these countries were calculated by dividing the number of reported suicides by the population size in the relevant age-band and sex group available on the same website. Data were ascertained for the latest five consecutive years. The one-year average suicide rate was calculated by dividing the sum of suicide rates for the latest five consecutive years by five. The median (range) for the latest year for the suicide rate data was 2005 (1983-2007).

The definition of elderly dependency ratio used in this study was the ratio of those over the age of 60 years to those under the age of 60 years. The age of 60 years was used as a cut-off age because the WHO provides figures for the proportion of the population over the age of 60 years. Data on the proportion of people over the age of 60 years and the total general population size was ascertained from the WHO website for the year 2006 (http://www.who.int/ countries/). From this the absolute numbers of people over the age of 60 years and under the age of 60 years in the general population was calculated. The elderly dependency ratio was calculated by dividing the number of people over the age of 60 years by the number of people under the age of 60 years. Child mortality rates (i.e. for those under the age of 5 years) were also ascertianed from the same WHO website to enable examination of the findings in the context of social development of countries as child mortality rates can be considered to be proxy for social development.

The relationship between elderly suicide rates, in both sexes in both the age-bands, and the elderly dependency ratios was examined by using Spearman’s correlation coefficient.

## Results

Full data set was available for 85 countries from the WHO website. A list of these 85 countries is provided in Table 1 along with their suicide rates, elderly dependency ratios and child mortality rates. The median (range) for suicide rates and elderly dependency ratios across the countries is provided in Table 2

**Table:** Table 1: **Median and range of suicide rates and elderly dependency ratios**

	Median	Range
Suicide rates males aged 65-74 years	19.6	0-93.98
Suicide rates males aged 75+ years	28.3	0-161.42
Suicide rate females aged 65-74 years	4.6	0-32.54
Suicide rate females aged 75+ years	5.06	0-71.36
Elderly dependency ratios	0.18	0.03-0.37
Suicide rates are per 100,000 of the relevant age and gender group		

**Table:** Table 2:**characteristics of the studied countries**

Country	Male 65-74 Suicide Rate	Male +75 Suicide Rate	Female 65-74 Suicide Rate	Female +75 Suicide Rate	Male Child Mortality Rate	Female Child Mortality Rate	Elderly Dependency Ratio
Albania	4.74	6.9	2.54	5.94	17	16	0.15
Antigua	0	0	0	0	13	10	0.12
Argentina	26.58	42.6	4.7	5.68	18	15	0.16
Armenia	7.34	7.12	1.76	5.06	26	21	0.16
Australia	19.6	25.58	4.92	4.56	6	5	0.22
Austria	50.06	87.98	13.76	19	5	4	0.28
Azebaijan	0	0	2.62	3.94	93	84	0.10
Bahamas	0	0	3.52	0	16	12	0.11
Bahrain	1.72	0	0	0	10	11	0.05
Belarus	86.12	76.4	15.26	20.4	63	75	0.22
Belgium	39.62	87.5	13.58	15.8	77	82	0.28
Belize	33.18	22.58	0	0	65	74	0.06
Bosnia	27.02	33.26	8.52	10.86	17	12	0.23
Brazil	12.52	14.6	2.38	2.28	22	18	0.10
Brunei	0	34.85	0	0	10	8	0.05
Bulgaria	38.78	54.34	15.56	28.35	13	12	0.3
Canada	17.48	21.56	4.66	3.3	6	5	0.22
Chile	27.62	34.64	2.42	3.16	10	8	0.14
Costa Rica	48.74	70.12	10.7	14.56	76	80	0.09
Croatia	60.86	99.70	17.94	24.64	72	79	0.28
Cuba	44.46	79.38	15.5	16.44	76	86	0.19
Czech Rep	31.74	66.8	8.31	15.42	5	3	0.25
Denmark	31.16	56.14	9.58	16.78	5	4	0.28
Dominica	0	0	0	0	16	14	0.12
Equador	10.52	12.52	1.36	1.98	70	76	0.1
El Salvador	15.44	17.06	0	0	28	23	0.09
Estonia	50.62	67.93	2.4	4.44	7	4	0.28
Finland	39.32	43.14	10.26	8.2	4	3	0.28
France	38.3	73.56	13.06	15.58	5	4	0.27
Georgia	14.34	12.96	3.20	6.08	33	31	0.22
Germany	28.72	56.26	10	16.88	5	4	0.33
Greece	7.88	13.12	1.78	1.74	4	4	0.3
Guatemala	6.34	6.08	0.72	0.9	41	41	0.06
Guyana	52.6	35.28	7.56	4.54	68	55	0.1
Hungary	68.08	121.36	18.66	33.72	8	6	0.27
Iceland	11.4	8.93	4.22	2.27	3	2	0.19
Ireland	14.16	9.28	4.22	2.8	5	4	0.18
Israel	15.28	28.08	4.74	8.14	6	5	0.15
Italy	18.5	33.56	5.5	6.42	4	4	0.35
Jamaica	0.88	0	0	0.4	33	30	0.11
Japan	42.4	45.02	18.62	23.4	4	3	0.37
Kazakhstan	61.5	60.88	11.68	20.94	4	3	0.11
Kiribati	0	0	0	0	65	63	0.06
Kuwait	0	0	0	0	12	10	0.03
Kyrgystan	24.28	25.34	4.26	7.62	44	38	0.08
Latvia	62.02	70.36	12.56	22.66	10	8	0.28
Lithiuania	87.56	84.38	19.92	25.56	9	8	0.27
South Africa	1.15	53.26	6.66	8.26	72	66	0.08
Spain	20.94	40.82	6.66	8.26	5	4	0.28
Surinam	14.55	44.91	4.6	0	44	33	0.1
Sweden	27	37.12	9.78	10.28	4	3	0.32
Switzerland	39.38	82.98	17.76	27.86	6	5	0.27
Tajikistan	6.2	4.46	2.22	1.68	74	61	0.05
Macedonia	23.96	49.84	12.44	19.10	19	15	0.19
Trinidad	28.5	32.6	5.24	11.58	39	36	0.11
Luxenberg	48.03	64.94	13.33	13.47	4	4	0.23
Maldives	0	0	0	18.2	34	27	0.06
Malta	19.72	16.55	4.81	6.32	7	5	0.23
Maritius	17.14	10.80	7.56	2.18	16	13	0.11
Mexico	9.34	15.8	1.04	0.94	38	32	0.10
Netherlands	16.7	29.4	7.7	8.42	6	5	0.25
New Zealand	17.34	26.5	4.52	4.88	7	6	0.2
Nicaragua	15.06	16.12	1.25	1.37	39	33	0.06
Norway	19.66	23.74	2.24	2.26	26	20	0.10
Panama	17.00	23.24	2.24	2.26	26	20	0.10
Paraguay	8.16	11.82	1.7	1.16	24	20	0.08
Peru	1.66	2.5	0	0	27	24	0.09
Poland	33.3	31.14	6.6	7.2	8	6	0.2
Portugal>	27.1	53.04	7.7	12.5	5	4	0.28
South Korea	93.98	161.42	8	27.38	5	5	0.16
Moldavia	42.24	37.94	11.36	10.48	23	15	0.18
Romania	30.72	29.3	7.7	6.42	18	15	0.23
Russia	80.76	86	15.56	11.56	15	11	0.2
St Kitts	19.03	0	0	0	18	20	0.12
St Lucia	20.26	47.49	0	13.91	14	14	0.11
St Vincents	0	57.15	0	0	24	16	0.1
Seychelles	26.01	17.57	0	0	12	13	0.11
Singapore	21.36	53.26	13.14	24.34	3	3	0.15
Slovakia	30.02	42.7	5.74	7	9	7	0.19
Slovenia	78.8	99.68	4.6	0	4	4	0.27

There were significant positive correlations between elderly dependency ratios and suicide rates in males aged 65-74 years (Rho=+0.60, P<0.0001), males aged 75+ years (Rho=+0.57, P<0.0001), females aged 65-74 years (Rho=+0.66, P<0.0001) and females aged 75+ years (Rho=+0.57, P<0.0001).

## Discussion

Some methodological issues need consideration. Cross-national data on suicide rates should be viewed cautiously because: data were not available from all countries^25,26^; the validity of this data was unclear^26,27^; the legal criteria for the proof of suicide vary between countries and in different regions within a country^26,28^ ; some countries; have poor death registration facilities^28^; and, cultural and religious factors and stigma attached to suicide may lead to under-reporting of suicides.^26,29^ The latest available data on suicide rates ranged from 1987 to 2007 and the elderly dependency ratios for several countries were from a different year, and these methodological issues may have biased the findings. Also, using a one-year average of five-year data on suicide rates does the denominator for calculating suicide rates, and this may have biased the findings. However, data was gathered from the WHO data bank and was the latest and best available cross-national data set, and a one-year average of five years data on suicide rates was used.

The significant positive correlations between suicide rates, in both sexes in both the elderly age-bands, and elderly dependency ratios confirmed the findings of the earlier study using only one-year cross-sectional data on elderly suicide rates. ^22^ The confirmation of earlier findings by using a more recent data set than the earlier study and one-year average of five years data on suicide rates suggests that the observed relationship between elderly suicide rates and elderly dependency ratios is accurate and robust. There may be several explanations for these findings. First, the findings may be an artifact of the methodological issues described above. Second, the findings were consistent with previous observations of positive correlations between elderly suicide rates and the proportion of elderly in the total population in large cross-national studies.^30,31^ This may reflect Durkheim’s hypothesis that the overall cohort size may influence suicide rates due to competition for scarce resources.^11^ Third, increased life expectancy was associated with increased suicide rates in the elderly in a cross-national study of 87 countries.^31^

Increased life expectancy leads to an increase in the proportion of elderly in the population.^31^ This may also reflect Durkheim’s hypothesis stated above. Fourth, an alternative hypothesis pertaining to cultural factors may be important and is explored below.

The impact of elderly dependency ratios on elderly suicide rates may interact with and be modified and/or mediated through cultural factors. These cultural factors were described in detail in the Introduction and include: the degree of respect and esteem given by younger generations to the elderly; the traditional dependence of elderly on their children for emotional and financial support and their children’s ability to provide this; the traditional expectation of the elderly to live with their children or grand-children; the greater effect on the elderly of their children’s negative attitudes; migration of children to urban areas or to other countries; and, the number of available caregivers, household size and family size. Countries with lower elderly dependency ratios would potentially have a greater number of younger people potentially available to positively contribute to these cultural issues, and this may ultimately lead to reduction in elderly suicide rates.

This study merely examined the relationship between elderly suicide rates and elderly dependency ratios and cultural factors were not formally measured. Therefore, considerable caution should also be exercised in assuming that elderly dependency ratios act as a proxy measure for cultural factors.

 Moreover, elderly dependency ratios may interact with, modify and mediate the effect of other factors on elderly suicide rates. An important factor in this context is the contribution of a greater number of younger people to the socio-economic status of countries because elderly suicide rates are also influenced by the socio-economic status of countries.^30,31^ The cross-sectional design of the study does not allow definitive conclusions about the aetiological relationship.

 The contribution of cross-national differences in cultural factors on elderly suicide rates requires further study by formally measuring: (i) the cultural views and attitudes of young people towards the elderly12; and (ii) the perception of the elderly on their traditional and changing role in society and their relationship with younger generations.
